# Scarification of the Gums—Reasons for It

**Published:** 1845-01

**Authors:** 


*Scarification of the Gums—Reasons for it.*—Dr. Marshall Hall says that there is no practical fact, of the truth and value of which he is more satisfied than that of the effect and efficacy of scarification of the gums in infants, and not in infants only, but in children. "The process of teething," he observes, "is one of augmented arterial action and of vascular action generally; but it is also one of augmented nervous action; for formation, like nutrition, secretion, &c., generally, is always one of nervi-vascular action, and of this the case in question is, from its peculiar rapidity, one of the most energetic. Like other physiological processes, it is apt to become, from that very character of energy, pathological, or of morbid activity. It is obviously, then, attended with extreme suffering to the little patient; the brain is irritable, and the child is restless and cross; the gums are tumid and heated; there is fever, an affection of the general vascular system, and there are, too frequently, convulsions of various degrees and kinds, manifested in the muscles which move the eyeball, the thumb and finger, the toes; the larynx, the parietes of the respiratory cavities; and the limbs and frame in general, affections of the excito-motor part of the nervous system, and of the secretions of the liver, kidneys, and intestines, affections of the ganglionic division of that system.

"What is the precise cause and source of these formidable effects? Can the mere tension and irritation of the gum situated over the more prominent part of the teeth be the cause of such extensive morbid actions? I think not. The real source of these phenomena is in the entire dental system, in which actions of unusual energy and extent are going on—sub-inflammatory they might be called, were they not, in reality, of an essentially different nature and origin. This undue action takes place in the fangs and sockets of the teeth in their whole extent, with their connections, vascular, nervous, and membranous. But *the* focus from which the *nervous* actions emanate is, I believe, not as is generally imagined, the nerves of the mere *gums* seated over the prominent parts of the teeth, but the nerves which may be emphatically termed *the nerves of the teeth themselves*, the nerves which enter into the very fangs and substance of the teeth.

"*It is to the base of the gums, not to their apex merely, that the scarification should be applied.* The most marked case in which I have observed the instant good effect of scarification was one in which *all the teeth had pierced the gums!*

"This view of the subject may assist in removing the futile objections of some who have, without due consideration I am convinced, opposed my plan of frequent, often daily, scarification of the gums, to whom I would say, as my sole reply: Better scarify the gums *unnecessarily* one hundred times than allow the accession of one fit or convulsion from the neglect of this operation, which is equally important in its results and trifling in its character.

"And it is not merely the prominent and tense gum over the edges of the teeth which should be divided; the gums, or rather the blood-vessels, immediately over the very *nerves of the teeth*, should be scarified and divided, as you would divide the vessels of the conjunctiva in inflammation of that membrane.

"Now, whilst there is fever or restlessness, or tendency to spasm or convulsion, this *local blood-letting* should be repeated daily, and in urgent cases even twice a day. I would here repeat my maxim: Better do this one hundred times unnecessarily than have one single fit from the neglect of so trifling an operation. A skilful person does it in a minute, and in a minute often prevents a most serious attack—an attack which may cripple the mind or limbs, or even take the life of our little patient, if frequently repeated. There is, in fact, no comparison between the means and the end, the one so trifling, the other so momentous."

*London Lancet*, May 18, 1844.

